# Oncologic and Reproductive Outcomes of Uterine Smooth Muscle Tumor of Uncertain Malignant Potential: A Single Center Retrospective Study of 67 Cases

**DOI:** 10.3389/fonc.2020.00647

**Published:** 2020-05-14

**Authors:** Lanqing Huo, Dan Wang, Wenze Wang, Dongyan Cao, Jiaxin Yang, Ming Wu, Junjun Yang, Yang Xiang

**Affiliations:** ^1^Department of Obstetrics and Gynecology, Peking Union Medical College Hospital, Chinese Academy of Medical Sciences and Peking Union Medical College, Beijing, China; ^2^Department of Pathology, Peking Union Medical College Hospital, Chinese Academy of Medical Sciences and Peking Union Medical College, Beijing, China

**Keywords:** smooth muscle tumor, uncertain malignant potential, surgical procedure, recurrence, reproductive outcomes

## Abstract

**Background:** The term “uterine smooth muscle tumor of uncertain malignant potential” (STUMP) indicates a rare tumor that cannot be classified as a benign leiomyoma or malignant leiomyosarcoma. In this study, we assessed the clinical characteristics, fertility, and oncologic outcomes of patients diagnosed as STUMP in 14 years. In addition, we analyzed the risk factors for recurrence in patients with STUMP.

**Methods:** Medical records of STUMP patients at Peking Union Medical College Hospital (PUMCH) between January 2005 and June 2019 were reviewed and analyzed. Disease-free survival, age of diagnosis, tumor size, surgical procedure, pathology and immunohistochemistry, clinical characteristics, recurrence rate, and reproductive outcomes in the follow-up period were assessed. Univariate and multivariate analyses were performed to determine the prognostic factors.

**Results:** The median age was 42 years old (range: 21–63). Total hysterectomy with or without bilateral salpingo-oophorectomy was performed in 29/67 cases (43.3%), and myomectomy was performed in 38/67 cases (56.7%). Ten patients experienced recurrences, and all but two recurrences occurred within 5 years after the initial surgery. Only two of these recurrences were leiomyosarcoma. There were no deaths in the median follow-up period of 48.4 (range 2.6–170.2) months. There were no remarkable differences in location of tumor between the myomectomy and hysterectomy groups, but the patients in the myomectomy group were younger than those in the hysterectomy group. In univariate and multivariate analysis, mitosis on pathology was the only independent risk factor for recurrence. Expression of Ki-67, p53, and p16 was significantly higher in patients with recurrence. Seven of the 35 patients who attempted to conceive had successful pregnancies.

**Conclusions:** The prognosis of STUMP was favorable and tumors with more than 10 mitoses per 10 high power field should be monitored closely. The surgical procedure was not an independent risk factor of recurrence, and myomectomy may be an acceptable option for patients wishing to preserve fertility.

## Introduction

Benign uterine leiomyomas, or smooth muscle tumors (SMT), are neoplasms of the myometrial layer of the uterus and are the most common tumor in pelvic of women (1). Less commonly, women with a uterine mass presumed to be a leiomyoma are found to have a uterine sarcoma or a leiomyoma variant. Leiomyosarcoma, a rare SMT subtype, has a significant contribution to uterine cancer deaths, and less than 30% of patients can hardly survive for 5 years after diagnosis (2). There are a number of other leiomyoma variants in which the SMT manifests one histologic facet typical of malignant neoplasm yet lacks others. These were called uterine smooth muscle tumors of uncertain malignant potential (STUMP) (3). The rarity of STUMP results in few analyses of its oncologic prognosis and fertility outcomes, and these studies are in very limited populations. Further, there are no available guidelines regarding the necessity of hysterectomy in young patients who may want to preserve their fertility. As far as we know, this retrospective study includes the largest number of STUMP cases reported so far. We present a comprehensive analysis of the clinical characteristics, oncologic outcomes, and fertility outcomes, intending to find an acceptable treatment for reproductive age women with STUMP and identify the risk factors for relapse.

## Materials and Methods

A retrospective review of medical records was conducted for Peking Union Medical College Hospital (PUMCH) patients seen between January 2005 and June 2019 with a pathologic diagnosis of STUMP. This study was approved by the institutional ethics committee of PUMCH (No. S-K929). The diagnosis criteria were first utilized in a previous clinico-pathological study of 213 uterine tumor cases (3). The inclusion criteria were modeled from the World Health Organization 2014 guidelines for the classification of STUMP (4). STUMP diagnosis in this study was based on meeting two or more of these criteria according to a study of pathology in STUMP in 2018 (5): (1) tumors with ambiguous necrosis; (2) tumors with more than two focal atypia and 8–9 mitoses/10 high power field (HPF) or with diffuse atypia; (3) tumors with a mitotic index of 15/10 HPF; (4) tumors with ischemic or coagulative necrosis in irregular shape or multifocal foci; (5) tumors with myxoid SMT and epithelioid morphology showing atypia or hyperproliferation; (6) myometrial invasion without malignant histological features; (7) atypical mitotic without malignant histological features.

Patients were divided into myomectomy and hysterectomy groups. The former included open, laparoscopic, and hysteroscopic myomectomy while the latter was made up of open and laparoscopic hysterectomy. Data were compared for the age at diagnosis, clinical characteristics, tumor size, surgical procedure, pathology, recurrence rate, disease-free survival (DFS), and fertility in the follow-up period. During the follow-up period, patients were recommended to undergo pelvic ultrasound every 6 months and abdominal-pelvic computed tomography (CT) scan every 2 years. The clinical characteristics were judged mainly by descriptive symptoms, like the compression of adjacent organs, menstrual irregularities, and pelvic pain. Serum cancer antigen 125 (CA-125) levels were also analyzed. Tumor size was analyzed as the largest measured diameter. The number of tumor was defined as the number of STUMP in one patient and multiple tumors mean more than one STUMP in the same patient. Pathology was analyzed as necrosis, mitoses number per 10 HPF, and atypical cells, as assessed by two experienced pathologists. Results were compared, and any discrepancies were resolved by discussion between the two pathologists. Immunohistochemistry stains were available for 26 cases, with assessments of Ki-67, p16, p53, estrogen receptor (ER) and progesterone receptor (PR) expression. Protein expression differences between the recurrence and non-recurrence groups were analyzed.

SPSS software version 24.0 (IBM Corporation, Armonk, NY, USA) for Macintosh was used for statistical analysis. Associations between clinical and pathologic characteristics and oncologic outcomes were analyzed. DFS was defined as the number of months between the date of initial surgery and the date of recurrence or censoring. Duration of follow-up and other descriptive statistics were identified as a number of patients and median, maximum and minimum of numeric variables. Mann-Whitney non-parametric *U*-tests were performed to compare median values. χ^2^ and Fisher's exact tests were performed to compare frequency distributions. Univariate analyses and multivariate analyses were performed by COX analysis to evaluate the risk factors for recurrence. Variables with *P* ≤ 0.2 on univariate analysis were selected for multivariate analysis for avoiding omitting the specific risk factors. Statistical significance was represented as *P* < 0.05.

## Results

### Clinical Characteristics

Records and follow up data were available for 67 patients. The median age of the patients was 42 years old (range 21–63) (Table 1). Six patients were postmenopausal, while all others were premenopausal at the time of pathologic diagnosis. Forty-three (64.2%) patients had compression of adjacent organs detected on physical exam. Twenty-nine (43.3%) patients had a complaint of menstrual irregularities, and nine patients had pelvic pain (13.4%). The median maximum tumor size was 7 (range 2–25) cm. There were 61 (91.0%) patients with more than one STUMP, but no leiomyomas, and only six patients had a single STUMP. The median serum CA-125 was 20.6 U/mL, ranging from 3.7 to 418.0 U/mL. All patients had transvaginal color Doppler imaging. Abdominopelvic CT was performed in 38 patients, while the remaining 29 patients had abdominopelvic magnetic resonance imaging (MRI) before surgery. In consideration of preserving fertility, 38 patients underwent myomectomy. There were 29 patients treated with total hysterectomy with or without bilateral salpingo-oophorectomy. The median follow-up time of all patients was 48.4 months (range 2.6–170.2).

**Table 1:** General clinical characteristics of patients (*n* = 67).

**Characteristics**	**Values**
Age (median, years)	42 (21–63)
Tumor size (median, cm)	7 (2–25)
Tumor no.	
Single	6 (9.0%)
Multiple	61 (91.0%)
Serum CA-125 (median, U/mL)	20.6 (3.7–418.0)
Surgical procedure	
Abdominal myomectomy	23 (34.3%)
Laparoscopic myomectomy	12 (17.9%)
Hysteroscopic myomectomy	3 (4.5%)
TAH+BSO/USO	16 (23.9%)
LH+BSO/USO	12 (17.9%)
CRS	1 (1.5%)
Mitosis	
<5	24 (35.8%)
5–10	35 (52.3%)
>10	8 (11.9%)
Necrosis	
Absent	52 (77.6%)
Focal/multifocal	15 (22.3%)
Atypia	
Mild	44 (65.7%)
Mild to moderate	14 (20.9%)
Moderate	8 (11.9%)
Moderate to severe	1 (1.5%)
Follow-up (median, months)	48.4 (2.6–170.2)

*TAH+BSO/USO, total abdominal hysterectomy + bilateral salpingo-oophorectomy/unilateral salpingo-oophorectomy; LH+BSO/USO, laparoscopic hysterectomy + bilateral salpingo-oophorectomy/unilateral salpingo-oophorectomy; CRS, cytoreductive surgery*.

### Pathological Characteristics

All pathology slides were reviewed by two experienced pathologists according to the classification of STUMP in the World Health Organization. Results were classified into three subtypes based on mitotic count per 10 HPF: 0–4 mitoses (*n* = 24), 5–10 mitoses (*n* = 35), and > 10 mitoses (*n* = 8) (Table 1). Focal or multifocal necrosis islands were seen in only six tumors. Results were further classified by diffuse or multifocal atypia as mild (*n* = 44), mild to moderate (*n* = 14), moderate (*n* = 8), and moderate to severe (*n* = 1) subtypes. Immunohistochemistry examinations were performed only in 26 patients (Table 2). The expression of biomarkers P16 and p53 in recurrent patients were much higher than in non-recurrent patients (*P* = 0.001; *P* = 0.01, respectively). The expression of Ki-67 was a very slight trend toward significance in the recurrent patients than in the non-recurrent patients (median 8 vs. 5%; *P* = 0.2).

**Table 2:** Clinical-pathological characteristics of patients who underwent Immunohistochemistry analysis (*n* = 26).

**Characteristics**	**Non-recurrence (*n* = 20)**	**Recurrence (*n* = 6)**	**t/χ^2^**	***P*-value**
Age (median, years)	43 (25–63)	28 (22–45)	–	0.03
Gravida	1 (0–4)	0.5 (0–4)	–	0.8
Parity	1 (0–2)	0.5 (0–1)	–	0.6
Tumor size (median, cm)	7.5 (2–17)	6.5 (3–15)	–	1
Follow-up (median, months)	33.2 (3.0–122.3)	68.9 (31.0–110.1)	–	0.05
Serum CA-125 (median, U/mL)	19.5 (7–418)	30.5 (7–70)	–	0.4
Tumor localization			1.2	0.5
Intramural	14 (70%)	5 (83.3%)	–	–
Subserous	2 (10%)	0 (0%)	–	–
Submucous	4 (20%)	1 (16.67%)	–	–
Immunohistochemistry				
Ki-67 (%)	5 (1–20)	8 (3–30)	–	0.2
P16 (+)	1 (5%)	4 (66.7%)	–	0.001
P53 (+)	3 (15%)	4 (66.7%)	–	0.01
ER (+)	4 (20%)	1 (16.7%)	–	0.9
PR (+)	4 (20%)	1 (16.7%)	–	0.9

*ER, estrogen receptor; PR, progesterone receptor*.

### Oncologic Outcomes

As shown in Table 3, there was a significant difference in age between the myomectomy and hysterectomy groups (33 vs. 47; *P* < 0.001), as well as the percentage of nulliparous women (22 vs. 2; *P* < 0.001), but not in the median interval between recurrence and diagnosis (49.3 vs. 45.1 months; *P* = 0.4). Furthermore, there was a significant difference between the two groups concerning the median tumor size (6.5 vs. 8.0 cm; *P* = 0.04) while there was no difference in the location of the tumor. There were 10 recurrent patients, and all, but two recurrences occurred within 5 years after the initial surgery. One of these patients underwent a hysterectomy at the initial treatment, while the other nine underwent myomectomy.

**Table 3:** Clinical characteristics and patient outcomes by surgery procedure (*n* = 67).

**Characteristics**		**Myomectomy (*n* = 38)**	**Hysterectomy (*n* = 29)**	**t/χ^2^**	***P-*value**
Age (median, years)		33 (21–52)	47 (26–63)		<0.001
Parity	Nullipara	22 (57.9%)	2 (6.9%)		<0.001
	Multipara	16 (42.1%)	27 (93.1%)		
Tumor size (median, cm)		6.5 (2–15)	8.0 (2–25)		0.04
Tumor no.	Single	5 (13.2%)	1 (3.4%)	1.9	0.2
	Multiple	33 (86.8%)	28 (96.6%)		
Uterine localization	Intramural	26 (68.4%)	19 (65.6%)	0.7	0.7
	Subserous	8 (21.1%)	5 (17.2%)		–
	Submucous	4 (10.5%)	5 (17.2%)		–
Surgical approach	Morcellation	13 (34.2%)	0 (0%)		
	Non-morcellation	25 (65.8%)	29 (100%)		
Follow-up (median, months)		49.3 (2.6–170.2)	45.1 (3.0–103.1)		0.4
Serum CA-125 (median,U/mL)		22 (7–79)	18 (4–418)		0.2
Recurrent pathology	STUMP	8 (21.1%)	0 (0%)	3.7	0.05
	LMS	1 (2.6%)	1 (3.4%)		

*Data shown are median (minimum-maximum) unless otherwise specified*.

*STUMP, uterine smooth muscle tumors of uncertain malignant potential; LMS, leiomyosarcoma*.

A comparison of different myomectomy surgical approaches is shown in Table 4A. The median tumor size in the laparoscopic and hysteroscopic myomectomy subgroup was 5.0 vs. 7.0 cm in the open myomectomy subgroup. A comparison of different hysterectomy surgical approaches is in Table 4B. The median tumor size in the laparoscopic subgroup was 6.0 vs. 9.0 cm in the open hysterectomy subgroup.

**Table 4A:** Characteristics and oncologic outcomes of patients with myomectomy (*n* = 38).

**Characteristics**		**Non-open (*n* = 15)**	**Open (*n* = 23)**	**t/χ^2^**	***P-*value**
Age (median, years)		37 (22–55)	29 (21–49)	–	0.08
Gravida		1 (0–2)	0 (0–4)	–	0.1
Parity		1 (0–1)	0 (0–1)	–	0.1
Tumor size (median, cm)		5.0 (2–10)	7.0 (3–15)	–	0.01
Tumor no.	Single	1 (6.7%)	4 (17.4%)	1	0.3
	Multiple	14 (93.3%)	19 (82.6%)	–	
Uterine localization	Intramural	9 (60%)	17 (74.0%)	2.4	0.3
	Subserous	3 (20%)	5 (21.7%)	–	
	Submucous	3 (20%)	1 (4.3%)	–	
Surgical approach	Morcellation	13 (86.7%)	0 (0%)	30.3	<0.001
	Non-morcellation	2 (13.3%)	23 (100%)		
Follow-up (median, months)		50.1 (10.6–130.7)	48.4 (2.6–170.2)	–	1.0
Recurrent pathology	STUMP	2 (13.3%)	6 (26.1%)	2.5	0.1
	LMS	1 (6.7%)	0 (0%)	–	

*Non-open, laparoscopic myomectomy and hysteroscopic myomectomy; Open, open myomectomy; STUMP, uterine smooth muscle tumors of uncertain malignant potential; LMS, leiomyosarcoma*.

**Table 4B:** Characteristics and oncologic outcomes of patients with hysterectomy (*n* = 29).

**Characteristics**		**Non-open (*n* = 12)**	**Open (*n* = 17)**	**t/χ^2^**	***P-*value**
Age (median, years)		45 (34–52)	49 (26–63)	–	0.07
Gravida		2 (1–5)	2 (0–4)	–	0.7
Parity		1 (1–2)	1 (0–2)	–	0.5
Tumor size (median, cm)		6 (2–13)	9 (5–25)	–	0.03
Tumor no.	Single	0 (0%)	1 (5.9%)	1	0.3
	Multiple	12 (100%)	16 (94.1%)		
Uterine localization	Intramural	8 (66.7%)	11 (64.7%)	1.7	0.4
	Subserous	1 (8.3%)	4 (23.5%)		
	Submucous	3 (25%)	2 (11.8%)		
Follow-up (median, months)		55.2 (3.0–103.1)	36.7 (4.2–99.0)	–	0.9
Recurrent pathology	STUMP	0 (0%)	0 (0%)	–	–
	LMS	1 (8.3%)	0 (0%)	–	–

*Non-open, laparoscopic hysterectomy and hysteroscopic hysterectomy; Open, open hysterectomy; STUMP, uterine smooth muscle tumors of uncertain malignant potential; LMS, leiomyosarcoma*.

The Clinico-pathological characteristics of recurrent patients are presented in Table 5. In the nine patients with recurrence after myomectomy, pathology revealed STUMP in eight cases and leiomyosarcoma in one case. The patient with recurrence after hysteroscopic myomectomy had leiomyosarcoma. The only recurrent patient in the hysterectomy group had leiomyosarcoma, and she underwent laparoscopic hysterectomy during the initial surgery. All 10 patients underwent a second surgery. The interval time between the initial surgery and the second surgery ranged from 3 to 65 months, with a median of 42.5 months. Eight of the recurrent tumor located in the uterus and two were pelvic masses. The patients with pelvic mass underwent cytoreductive surgery. One patient with recurrence in the uterus underwent myomectomy, while the remaining seven patients underwent total abdominal hysterectomy with or without bilateral salpingo-oophorectomy. The median follow-up time was 6 years, ranging from 8 to 170 months. All recurrent patients were alive with no evidence of disease at the last follow-up.

**Table 5:** Clinico-pathological characteristics of patients with recurrent disease (*n* = 10).

			**Initial treatment**								**Recurrent treatment**				
**No**	**Age (year)**	**Parity**	**Size (cm)**	**Morcellation**	**Surgery**		**P16**	**P53**	**Ki-67**	**Interval time (months)**	**Location**	**Surgery**	**Pathology**	**Outcome**	**Follow up (months)**
1	22	0	3	No	HM	Hys	+	+	+	12	Uterus	TAH+BSO	LMS	ANED	50
2	28	0	3	No	AM	Open	–	–	–	38	Uterus	TAH	STUMP	ANED	170
3	45	1	15	No	AM	Open	+	–	+	50	Pelvic mass	CRS	STUMP	ANED	110
4	24	0	5	No	AM	Open	–	+	–	47	Uterus	AM	STUMP	ANED	72
5	35	1	8	No	LH+BSO	Lap	+	+	+	6	Pelvic mass	CRS	LMS	ANED	72
6	28	0	10	No	AM	Open	–	+	+	65	Uterus	TAH+BSO	STUMP	ANED	66
7	44	0	10	No	AM	Open	–	–	+	3	Uterus	TAH+BSO	STUMP	ANED	8
8	28	1	5	Yes	AM	Lap	+	–	+	17	Uterus	TAH+BSO	STUMP	ANED	31
9	49	1	10	No	AM	Open	–	–	–	60	Uterus	TAH+BSO	STUMP	ANED	91
10	40	1	10	Yes	AM	Lap	–	–	–	62	Uterus	TAH+USO	STUMP	ANED	131

*HM, hysteroscopic myomectomy; Hys, hysteroscopic; AM, abdominal myomectomy; Open, open abdominal approach; LH+BSO, laparoscopic hysterectomy with bilateral salpingo-oophorectomy; Lap, laparoscopic approach; TAH: total abdominal hysterectomy; TAH+BSO: total abdominal hysterectomy with bilateral salpingo-oophorectomy; TAH+USO: total hysterectomy with unilateral salpingo-oophorectomy; CRS, cytoreductive surgery; LMS, leiomyosarcoma; STUMP, uterine smooth muscle tumors of uncertain malignant potential; Interval time, interval between initial surgery and recurrence; ANED, alive with no evidence of disease*.

**Figure 1:**
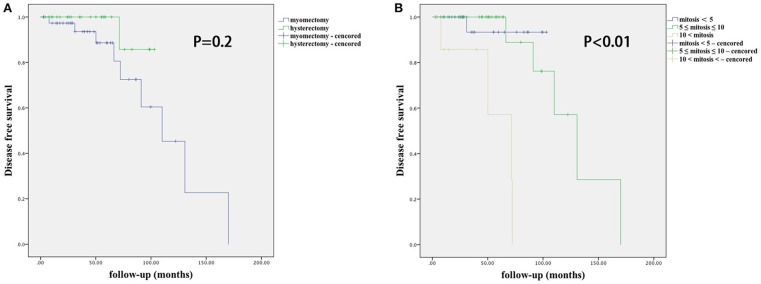
Disease-free survival according to surgical procedure **(A)** and mitosis number **(B)**.

The risk factors for recurrence in patients with STUMP are shown in Table 6 and Figure 1. In univariate analysis, age, parity, tumor size, number, location, serum CA-125, surgical approach, and necrosis were not significantly associated with recurrence. Recurrence was significantly associated with mitosis in the initial pathology (*P* = 0.003, 95% confidential interval, 95% CI = 2.2–212.3), and to a lesser degree with surgical procedure type (*P* = 0.2, 95% CI = 0.5–32.9) and tumor locations (*P* = 0.2, 95% CI = 0.06–4.8). After multivariate analysis, mitosis in the initial pathology was the only independent risk factor for recurrence (*P* = 0.007). There was no significant difference in DFS between different surgical procedure groups (*P* = 0.2), nor in different tumor location groups (*P* = 1).

**Table 6:** Univariate and multivariate analysis of risk factors for recurrence (*n* = 67).

	**Univariate**		**Multivariate**	
**Characteristics**	**HR 95% CI**	***P*-value**	**HR 95% CI**	***P*-value**
Age (y)		0.7		
<42	1			
≥42	0.8(0.2–3.3)			
Parity		0.6		
Nullipara	1			
Multipara	0.7(0.2–2.5)			
Tumor size (median, cm)		0.7		
<7	1			
≥7	1.3(0.3–5.4)			
Tumor no.		0.8		
Single	1			
Multiple	0 (0.1–>1,000)			
Tumor location		0.2		1
Submucous	1		1	
Subserous	0.5 (0.06–4.8)	0.6	1 (0.09–10.7)	1
Intramural	0 (0–>1,000)	1	0 (0–>1,000)	1
CA-125(median, U/mL)		0.8		
<20.6	1			
≥20.6	1.2 (0.3–5.2)			
Surgical procedure		0.2		0.2
Hysterectomy	1		1	
Myomectomy	4.0 (0.5–32.9)		3.8 (0.4–33.0)	
Surgical approach		0.6		
Laparoscopic or hysteroscopic	1			
Open	0.7(0.2–2.5)			
Morcellation		0.9		
No	1			
Yes	0.9 (0.2–4.6)			
Mitosis		0.003		0.007
<5	1		1	
5–10	1.3 (0.1–14.1)	0.9	1.4 (0.1–16.3)	0.8
>10	21.6 (2.2–212.3)	0.008	19.2 (1.9–190.9)	0.01
Necrosis		0.8		
Absent	1			
Focal/multifocal	0.8 (0.09–7.2)			
Atypia		0.9		
Mild	1			
Mild to moderate	0.6 (0.1–4.0)	0.6		
Moderate	1.8 (0.2–15.9)	0.6		
Moderate to severe	0.7 (0.05–9.6)	0.8		

*HR, hazard risk; 95%CI, 95% confidential interval*.

### Reproductive Outcomes

Among the 38 patients with myomectomy during initial treatment, 35 patients had a desire to achieve pregnancy, and the other three patients, who were over age 45, did not. Seven of the women (20.0%) conceived successfully and gave birth to seven healthy babies (Table 7). Three of them underwent total hysterectomy with bilateral salpingo-oophorectomy at the time of the cesarean section due to a desire to minimize the risk of recurrence. The follow-up time for these seven patients ranged from 16 to 86 months. At the last follow-up, all patients were alive without evidence of disease.

**Table 7:** Clinical information of patients with myomectomy who had successful pregnancies (*n* = 7).

**No**.	**Age (year)**	**Location**	**Size (cm)**	**Tumor no**	**Initial surgery**	**Interval (month)**	**Later surgery**	**Outcome**	**Follow up (month)**	**Outcome**
1	28	Subs	6	Single	AM	16	CS	Live birth	24	ANED
2	29	Subm	7	Multiple	AM	25	CS	Live birth	86	ANED
3	34	Subs	5	Single	AM	17	CS+TAH+BSO	Live birth	51	ANED
4	29	Intra	8	Single	AM	16	CS+TAH+BSO	Live birth	16	ANED
5	27	Intra	5	Multiple	AM	20	CS	Live birth	29	ANED
6	31	Intra	7	Multiple	AM	21	CS+TAH+BSO	Live birth	45	ANED
7	21	Intra	7	Multiple	AM	34	CS	Live birth	60	ANED

*Subs, subserous; subm, submucous; intra, intramural; AM, abdominal myomectomy; CS, cesarean section; TAH+BSO, total hysterectomy with bilateral salpingo-oophorectomy; Interval time, time between initial and later surgery; ANED, alive with no evidence of disease*.

## Discussion

These findings present several exciting and meaningful results. The first concern was the choice of surgical procedure for STUMP patients of reproductive age. According to the limited statistics, STUMP is generally found in younger aged women than other tumors of the uterus. In this study, the median age of patients was 42 years, similar to previously reported cases (6–12). Since the patients are young, preserving fertility is an essential consideration in the selection of treatment. According to a review published in 2019 (13), surgery is the standard treatment for STUMP. Although there are no treatment guidelines for STUMP, at present, the common practice is hysterectomy with or without bilateral salpingo-oophorectomy if patients have no fertility desire (5, 6, 8, 9). For patients with fertility desire, myomectomy should be considered as the first-line treatment (13). There are several reported cases of successful pregnancy after myomectomy for STUMP (14). In this study, 35 patients who underwent myomectomy and were ≤45 years old attempted to conceive during the follow-up period, and 7 (20.0%) succeeded in achieving seven singleton pregnancies. All of these patients underwent a cesarean section to reduce the risk of uterine rupture with vaginal delivery after myomectomy. Similarly, previously reported patients with successful childbirth after myomectomy were young, and most of them had cesarean sections (14). Patients in this study and previous studies all had a regular follow-up to assess for relapse while attempting to conceive. As stated by Angiolo and Gian (13), an accurate assessment should be provided to rule out the probability of recurrence before attempted pregnancy. Also, a delayed hysterectomy is recommended for patients with completed childbearing (13). Based on the results of this study, however, the necessity of hysterectomy after childbirth should be considered carefully. During the 16 to 86 months follow-up period, all patients survived without evidence of disease, including four who underwent cesarean section without a total hysterectomy and bilateral salpingo-oophorectomy. The prognosis was favorable for patients in this study. This message is good news for women who want to preserve their fertility, since delaying hysterectomy until recurrence could be safe.

The second important finding concerns the effect of surgical approaches and regular follow-up on recurrences. There are no previous reports about the effect of the surgical approach on disease recurrence in STUMP. Laparoscopic and open abdominal approaches may be used in both myomectomy and hysterectomy. Morcellation could be used in both myomectomy and hysterectomy, and this may cause metastasis (15). In this study, there were no morcellation in open cases. In the comparison of open and laparoscopic or hysteroscopic approaches in our study, there was no noticeable difference in recurrence rates (Table 6). The excellent prognosis seen in this study may be in connection with regular follow-up. Periodical controls have been suggested in some studies, although there is no consensus regarding follow-up protocols (9). Recurrent tumors could be detected in a timely manner by CT or MRI. Patients in this study were followed according to a suggestion of 6-month intervals for repeat physical examinations and imaging tests (16). Since almost all recurrence happened within 5 years after initial surgery, this may be a guide for the minimum follow-up duration. While CA-125 is a useful marker for the prognosis and severity of ovarian cancers, it is not a reliable marker for STUMP, as there were minimal differences for CA-125 levels between the recurrence and non-recurrence groups.

The third important message is the pathological forecast for recurrences. It has previously been reported that the recurrence rate of STUMP ranged from 0 to 36.4%, and median interval time from initial treatment to recurrence was 51 months, ranging from 15 to 9 years (6–8, 12). Molecular studies of SMT and leiomyosarcoma have identified the potential expressed proteins associated with poor prognosis in STUMP (16). A recent study analyzed proteins p16, p53, Ki-67, estrogen receptor (ER), and progesterone receptor (PR) and illustrated that these proteins could be biomarkers for aggressive tissue (17). Further, expression of MIB-1, p53, and PR could be helpful for the differentiation of SMT, STUMP, and leiomyosarcoma (18–21). Since the prognosis of leiomyosarcoma is much worse than that for STUMP, it is vital to determine the risk factors for relapse and leiomyosarcoma. As shown in Table 5, both patients with leiomyosarcoma had an expression of proteins p16 and p53 in the immunohistochemical tests, indicating that we should pay more attention to the risk of leiomyosarcoma recurrence in those patients with expression of p16 and p53. Recently, immunohistochemical stains have been suggested in several studies to identify which patients with STUMP had high recurrent risk, and those markers including epithelial growth factor receptor, p53, p16, galectin-3, MIB-1, BCL-2, Twist, estrogen receptor (ER), and progesterone receptor (PR) (20–22). As with previous publications, this study indicates that proteins p16 and p53 may be markers for recurrence of STUMP, considering the remarkable differences between the non-recurrence and recurrence groups (*P* = 0.001; *P* = 0.01). Additionally, Ki-67 also may have an association with recurrence. It appears advisable to perform immunohistochemistry tests of proteins p16, p53, and Ki-67 in all STUMP patients. During a median follow-up period of 48.4 months (range 2.6–170.2 months), 10 patients experienced recurrences, but there were no deaths, and meaningful survival rate analysis was not possible. However, considering the recurrence pathology and the malignant characteristics of leiomyosarcoma, we should focus on finding the risk factors of recurrence. As discussed, histologists should add immunohistochemistry stains for p16, p53, and Ki-67 when they find a variety of mitosis in 10 HPF in the microscopic evaluation of STUMP. Since mitosis indicates cell migration activity and recurrence has a close connection with this activity, the more mitosis cells, the more probability for recurrence. What is more, long term follow-up monitoring, including gynecologic examinations and imaging, may be necessary for patients with STUMP.

Although this study includes the largest number of cases to date, the number of patients in this study is still limited and precludes a definitive conclusion concerning optimal surgical approaches. Further long-time follow-up may be needed to confirm the safety of myomectomy vs. hysterectomy and the safety of laparoscopic vs. open techniques. Additionally, the long-time frame of the investigation could contribute to weak pathologic consistency in the diagnosis of this rare tumor and reduced the number of patients with immunohistochemical results, which limits the evaluation of biomarkers. Additional studies are needed to confirm the value of prognostic biomarkers, including Ki-67, p16, and p53. What is more, this is a retrospective study, and some bias in statistics and analysis is possible.

## Conclusions

In STUMP, regular follow-up is suggested for evaluating the real impact of treatments, especially in the first 5 years. Expression of p16, p53, and Ki-67 may be risk factors for relapse. Immunohistochemical evaluation should be indicated for all patients. STUMP has a favorable prognosis, and for patients with a desire to preserve fertility, myomectomy and regular follow-up may be a safely alternative to hysterectomy.

## Data Availability Statement

The datasets for this article are not publicly available due to patient privacy concerns. Requests to access the datasets should be directed to Lanqing Huo, nancy-huo@foxmail.com.

## Ethics Statement

The studies involving human participants were reviewed and approved by the institutional ethics committee of Peking Union Medical College Hospital (No. S-K929). The patients/participants provided their written informed consent to participate in this study.

## Author Contributions

LH, DW, and YX: study conception and design. DC, JiY, and JuY: literature review and data acquisition. WW, MW, and JiY: quality control. LH and DW: statistical analysis and manuscript preparation. LH, DW, WW, DC, JiY, MW, JuY, and YX: manuscript review.

## Conflict of Interest

The authors declare that the research was conducted in the absence of any commercial or financial relationships that could be construed as a potential conflict of interest.
